# Supplementary material for: Deletion of wfs1 Impairs Oligodendrocyte Precursor Cells Dorsal Distribution and Myelination Through the wfs1-hmgcs1 Axis in Zebrafish
Source: Biology (Basel). 2026 Aug 16;15(16):1402. doi: 10.3390/biology15161402 (PMC13510151; doi:10.3390/biology15161402)
Supplement: Supplementary file 1 [file biology-15-01402-s001.zip › File S1-Transcriptome sequencing data and differentially expressed genes (DEGs) in wfs1-- zebrafish at 5 dpf/2.KEGG/wfs1KOvsWT/ALL/src/dre04137.html]

dre04137


- dre:336159

- Down regulated genes

336159\_1(-1.30651991896487)

- dre:555713

- Up regulated genes

555713(1.53554204240876)

- Down regulated genes

555713\_1(-1.96184994943554)

- dre:100000242

- Down regulated genes

100000242(-6.34512572021214)
- dre:326974

- Up regulated genes

326974\_1(1.07837684837292)

- dre:561933

- Up regulated genes

561933\_2(2.8695106368199) 561933\_1(1.83182094817085)

- Down regulated genes

561933(-3.22949295062847)

- dre:564996

- Up regulated genes

564996(1.35939178896065)

- dre:492676

- Up regulated genes

492676(1.45180580384652)

- dre:559564

- Down regulated genes

559564(-1.18025564761092)

- dre:556410

- Up regulated genes

556410\_1(1.7264669285964)

- dre:550134

- Up regulated genes

550134\_1(1.8446004641876)

- Down regulated genes

550134(-4.18593720111435)
- dre:777766

- Up regulated genes

novel.905(1.76535773054974)

- dre:550134

- Up regulated genes

550134\_1(1.8446004641876)

- Down regulated genes

550134(-4.18593720111435)
- dre:777766

- Up regulated genes

novel.905(1.76535773054974)

Close
